# Isoform‐dependent regulation of Tau function by phosphorylation

**DOI:** 10.1002/alz.085651

**Published:** 2025-01-03

**Authors:** Nima N Naseri, Taylor L Tomlinson, Marie Shimogawa, Ophir Shalem, E. James Petersson, Elizabeth Rhoades

**Affiliations:** ^1^ University of Pennsylvania, Philadelphia, PA USA; ^2^ Children’s Hospital of Philadelphia at University of Pennsylvania, Philadelphia, PA USA

## Abstract

**Background:**

Tau is a neuronal microtubule associated protein whose interactions with microtubules are regulated by phosphorylation. Tau has numerous putative phosphorylation sites, but it is unclear which combinations of Tau phosphorylation co‐occur in the normal state and precisely how they impact Tau function. Adding further complexity, there are six major Tau isoforms arising from alternative splicing. These isoforms are expressed in an age‐dependent manner: only 3R (3‐repeats in the microtubule‐binding domain) Tau is expressed in development, while both 3R and 4R (4‐repeats in the microtubule‐binding domain) Tau isoforms are present in adult brain.

**Method:**

We applied time course studies in mice, sub‐cellular MT fractionation from human iPSC‐derived neurons as well as biophysical assays using recombinant Tau phospho‐mimics and purified tubulin to investigate the isoform‐specific impacts of phosphorylation to Tau‐tubulin/MT interactions. Significantly, we semi‐synthesized Tau, modified with bonafide site‐specific phosphorylation and a fluorescently labeled unnatural amino acid, to measure the impact of phosphorylation in the proline‐rich region to tau‐tubulin binding at the single molecule level.

**Result:**

Our data reveal that in each of the platforms investigated, 3R Tau function is more dramatically altered by phosphorylation as compared to 4R Tau upon modification at the same residues. These isoform‐specific differences were most pronounced at phosphorylation sites in the proline‐rich region of Tau, and significantly less so in the C‐terminal tail.

**Conclusion:**

Our findings highlight the possibility of differential regulation of Tau isoforms that reflect different roles for Tau in the developing and adult brains. Overall, our findings provide insight into functional regulation of Tau‐tubulin/MT interactions by phosphorylation, which is critical for ultimately understanding the significant increase in phosphorylated Tau found in pathology and whether specific phospho‐Tau forms can be targeted for therapeutic intervention.

